# Comparison of Perioperative Outcomes of Robot-Assisted vs. Laparoscopic Radical Nephrectomy: A Systematic Review and Meta-Analysis

**DOI:** 10.3389/fonc.2020.551052

**Published:** 2020-09-18

**Authors:** Jinze Li, Lei Peng, Dehong Cao, Bo Cheng, Haocheng Gou, Yunxiang Li, Qiang Wei

**Affiliations:** ^1^Department of Urology, Nanchong Central Hospital, The Second Clinical Medical College, North Sichuan Medical College, Nanchong, China; ^2^Department of Urology, Institute of Urology, West China Hospital, Sichuan University, Chengdu, China; ^3^Department of Neurology, The Affiliated Hospital of Medical College, North Sichuan Medical College, Nanchong, China; ^4^Department of Otolaryngology, Nanchong Central Hospital, The Second Clinical Medical College, North Sichuan Medical College, Nanchong, China

**Keywords:** renal cell carcinoma, radical nephrectomy, robotic, laparoscopic, meta-analysis

## Abstract

**Background:** The use of robot-assisted radical nephrectomy (RARN) for renal cell carcinoma (RCC) has increased in recent years, but the advantages of RARN over laparoscopic radical nephrectomy (LRN) remain controversial. This study aimed to compare the perioperative outcomes between RARN and LRN.

**Methods:** We systematically searched the EMBASE, PubMed, Web of Science, and CNKI databases to identify eligible comparative studies. The parameters were perioperative outcomes including operating time (OT), estimated blood loss (EBL), length of stay (LOS), conversion rate, and complications. Stata 15.0 software was used for the meta-analysis.

**Results:** Seven studies with 1,832 patients were included in the analysis. Among them, 532 underwent RARN and 840 underwent LRN for RCC. There were no significant differences in OT (weighted mean difference [WMD], 29.05; 95% confidence interval [CI], −0.31, 58.41; *p* = 0.05), EBL (WMD, −4.56; 95% CI, −29.79, 20.67; *p* = 0.72), LOS (WMD, −0.34; 95% CI, −0.68, 0.00; *p* = 0.05), conversion rate (WMD, 2.67; 95% CI, 0.68, 10.46; *p* = 0.05), transfusion rate (odds ratio [OR], 1.30; 95% CI, 0.74, 2.27; *p* = 0.36), intraoperative complications (OR, 1.13; 95% CI, 0.61, 2.12; *p* = 0.62), and postoperative complications (OR, 1.07; 95% CI, 0.68, 1.67; *p* = 0.62) between the two groups.

**Conclusion:** RARN was not superior to LRN in patients with RCC in terms of perioperative outcomes. Before establishing conclusive clinical recommendations, high-quality prospective large-scale randomized controlled trials with long-term follow-up are needed.

## Introduction

Renal cell carcinoma (RCC) is the most common genitourinary malignancy, with estimated 403,262 new cases and 175,098 associated deaths worldwide in 2018 (1). Radical nephrectomy (RN) is the standard procedure for the management of large kidney tumors or tumors not suitable for nephron-sparing surgery (2, 3). In recent years, after the development of minimally invasive techniques, laparoscopic RN (LRN) has been considered an alternative to traditional open RN because it is associated with less trauma and fewer perioperative complications (4). However, laparoscopic technology has limited flexibility and operability, and its learning curve is steep (5).

In 2005, a new surgical procedure for RN was performed by Klingler et al. (6), known as robot-assisted radical nephrectomy (RARN). Since its introduction, RARN has been considered a safe and powerful procedure for nephrectomy by multiple institutions, and its use has increased significantly during the past decade (7). Robotic procedures have several significant advantages over laparoscopy, including higher definition displays, finer manipulations, and a greater range of motion (8).

Although the robotic procedure for RN has been increasingly adopted worldwide, its advantages in treating renal tumors are still controversial. Several studies have reported that RARN is associated with similar perioperative outcomes and higher hospital charges than the standard LRN (9–11). However, others have found that RARN is associated with lower surgical morbidity (12). This study aimed to perform a standard meta-analysis of the literature to compare the perioperative outcomes of RARN and LRN in patients with RCC.

## Methods

### Search Strategy

This meta-analysis was performed according to the PRISMA (Preferred Reporting Items for Systematic Reviews and Meta-Analysis) criteria (13) and was prospectively registered in the PROSPERO (CRD42020143279). We searched the EMBASE, PubMed, Web of Science, and CNKI databases for articles published between January 2005 and January 2020. The following search terms were used: “robot-assisted,” “robotic-assisted,” “robotic,” “robot,” “laparoscopic,” “laparoscopy,” “radical,” and “nephrectomy.” No language restriction was used. We also manually retrieved the reference list of eligible studies and reviewed conference records. Each included study was evaluated independently by two reviewers (J.L. and L.P.), and any differences were resolved by consensus.

### Inclusion/Exclusion Criteria

The following inclusion criteria were used: (1) study type was randomized controlled trial, cohort study, or case-control study; (2) studies performed in adults diagnosed with RCC; (3) studies comparing RARN with LRN; (4) evaluation of at least one perioperative outcome such as operating time (OT), estimated blood loss (EBL), length of stay (LOS), conversion rate, transfusion rate, and complications.

The exclusion criteria were as follows: (1) reviews, letters to editors, case reports, and unpublished articles; (2) no comparison performed between RARN and LRN; (3) patients with benign kidney tumors; (4) studies with unavailable or unclear data.

### Data Extraction

Data extraction was conducted independently by two authors (J.L. and L.P.), and disagreement was resolved through negotiation. The following data were extracted from eligible studies: first author, publication date, study type, surgical procedure, number of patients, age, body mass index, tumor size, follow-up time, and outcome measures (including OT, EBL, LOS, conversion rate, transfusion rate, and complications). If continuous variables in the article were expressed as median (interquartile range), we converted it to mean ± standard deviation (14).

### Risk-of-Bias Assessments

Publication bias was evaluated using the Risk of Bias in Non-Randomized Studies-of Interventions (ROBINS-I) tool (15). This tool assesses seven domains: confounding bias, selection bias, bias in measurement classification of interventions, bias due to deviations from intended interventions, bias due to missing data, bias in measurement of outcomes, and bias in selection of the reported result.

### Quality Assessment

The quality of all included studies was estimated using the Newcastle–Ottawa scale (maximum score 9) (16). A score of ≥6 was considered high quality, whereas a score of ≤ 5 indicated low quality. Additionally, the level of evidence for each study was appraised according to the evidence evaluation criteria published by the Oxford Evidence-based Medicine Center (17). Two reviewers (J.L. and L.P.) performed quality assessment and level of evidence on the included studies, and differences were resolved through negotiation.

### Statistical Analysis

The meta-analysis was performed using Stata v.15.0 software (Stata Corp, College Station, TX, USA). Continuous and dichotomous variables were pooled as weighted mean difference (WMD) and odds ratio (OR), respectively. All data were reported with 95% confidence intervals (CIs). The *Z* test was conducted to determine all merged effects, and statistical significance was defined as *p* < 0.05. Heterogeneity between studies was estimated using the χ^2^-test and inconsistency (*I*^2^) test; *p* < 0.10 or *I*^2^ > 50% indicated significant heterogeneity, and the random-effects model was applied; otherwise, a fixed-effects model was adopted. Sensitivity analysis was performed by omitting individual studies one by one for some outcomes such as OT, EBL, and LOS. Because fewer than 10 studies were included in this meta-analysis, no funnel plot was used to evaluate publication bias.

## Results

### Study Characteristics

**Figure 1:**
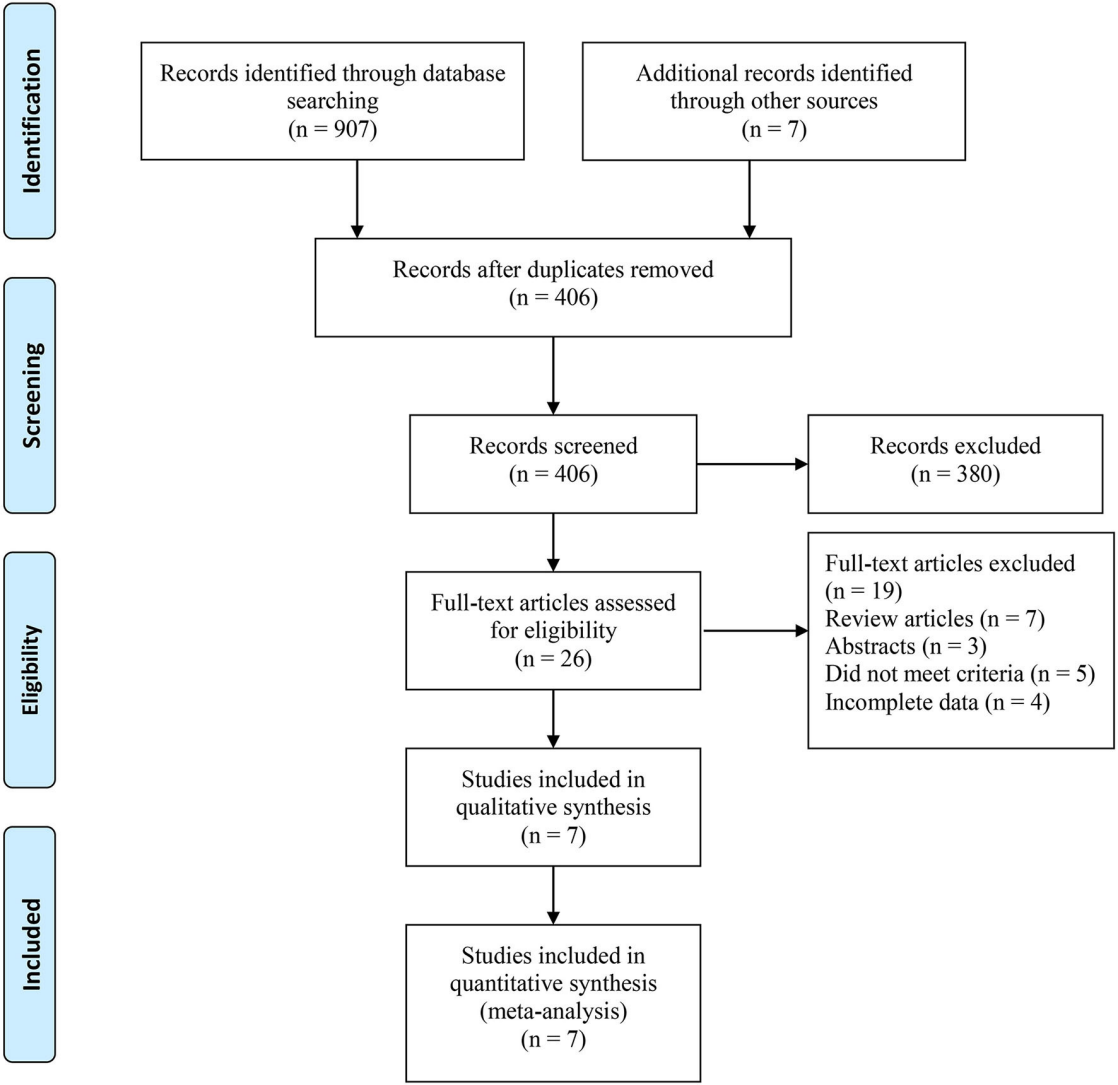
Flow diagram of studies identified, included, and excluded.

Based on the search strategy, we identified 914 related articles from the database. Among them, 406 studies remained after removing duplicates. After reading the title and abstract, 380 articles were excluded. Finally, seven full-text studies involving 1,832 patients (726 RARN vs. 1,070 LRN) were included in this meta-analysis (9, 10, 18–22) (Figure 1). Among the seven studies, there were three prospective (18–20) and four retrospective studies (9, 10, 21, 22). Only one study reported patient matching (10). The characteristics and level of evidence of all the included studies are presented in Table 1, and the quality evaluation of the included studies is presented in Table 2.

**Table 1:** Baseline characteristics of include studies and methodological assessment.

**Authors**	**Year**	**Study design**	**Surgical method**	**Patients (*N*)**	**Age (years)**	**BMI**	**Tumor size (cm)**	**Follow-up (months)**	**Quality score**	**Level of evidence**
Nazemi et al. (18)	2006	Prospective	RARN/LRN	6/12	63.2 ± 32.4/62.7 ± 27.7	27.1 ± 11.4/28.9 ± 17.3	4.3 ± 2.6/7.1 ± 10.7	4 (1–10)/7 (1–21)	7	4
Hemal et al. (19)	2009	Prospective	RARN/LRN	15/15	50.3 ± 10.2/52.7 ± 11.8	28.3 ± 4.5/29.1 ± 3.4	6.7 ± 2.3/6.9 ± 2.1	8.3 (1–12)/9.1 (2–12)	7	4
White et al. (9)	2011	Retrospective	RARN/LRN	10/10	66 ± 17.2/66.5 ± 11.2	29.4 ± 6/30.5 ± 8.2	5.5 ± 2.2/7± 2.9	10.5	7	2b
Helmers et al. (20)	2016	Prospective	RARN/LRN	76/243	62± 11.5/62.1 ± 13.1	27.8 ± 6.3/28.9 ± 5.4	5.6 ± 2.9/6 ± 3	NR	7	4
Golombos et al. (10)	2017	Propensity score match	RARN/LRN	230/230	73.7 ± 6.0/74.2 ± 6.2	NR	4.8 ± 2.6/4.8 ± 2.3	3.2^*^	7	2b
Li et al. (21)	2017	Retrospective	RARN/LRN	21/23	59.1 ± 13.4/59.4 ± 5.5	25.8 ± 3.5/24.8 ± 3.5	NR	5.1 (3–10)/3.9 (3–9)	6	4
Anele et al. (22)	2019	Retrospective	RARN/LRN	404/537	62.6 ± 12.3/63.3 ± 11.2	27.8 ± 4.7/26.9 ± 5.64.4	8.7 ± 2.1/8.9 ± 1.5	14.9 (6–34)/20.2 (7–43.2)	8	2b

*RARN, robot-assisted radical nephrectomy; LRN, laparoscopic radical nephrectomy; BMI, body mass index; NR, not report. ± refers to standard deviation, Numbers in brackets refers to the range*.

* *The mean follow-up of 3.2 years*.

**Table 2:** Quality assessment of included studies.

**Study**	**Selection**				**Comparability**		**Exposure**			**Total points**
	**REC**	**SNEC**	**AE**	**DO**	**SC**	**AF**	**AO**	**FU**	**AFU**	
Nazemi et al. (18)	1	1	1	1	1		1		1	7^*^
Hemal et al. (19)	1	1	1	1	1		1		1	7^*^
White et al. (9)	1	1	1	1	1		1		1	7^*^
Helmers et al. (10)	1	1	1	1	1		1		1	7^*^
Golombos et al. (10)	1	1	1	1	1		1		1	7^*^
Li et al. (21)	1	1	1	1	1		1			6
Anele et al. (22)	1	1	1	1	1		1	1		8^*^

*REC, representativeness of the cohort; SNEC, selection of the none posed cohort; AE, ascertainment of exposure; DO, demonstration that outcome of interest was not present at start of study; SC, study controls most important factors; AF, study controls for other important factors; AO, assessment of outcome; FU, follow-up long enough for outcomes to occur (“long enough” is defined as 1 year); AFU, adequacy of follow-up of cohort (≥ 80%)*.

* *Means that the study is satisfied the item, the quality score ≥7 points was ranked as high*.

### Demographic Variables

There were no significant differences between the two groups in terms of age (WMD, −0.55; 95% CI, −1.40, 0.30; *p* = 0.21), sex (male/total: OR, 1.00; 95% CI, 0.82, 1.22; *p* = 0.98), body mass index (WMD, 0.45; 95% CI, −0.07, 0.98; *p* = 0.09), and tumor size (WMD, −0.08; 95% CI, −0.28, 0.12; *p* = 0.43; Table 3).

**Table 3:** The demographics of the studies.

**Outcomes**	**No. of studies**	**No. of patients**	***P*-value**	**WMD or OR (95% CI)**	**Heterogeneity**			
		**RARN/LRN**			**Chi^**2**^**	**df**	***P***	***I*^**2**^ (%)**
Age	7	762/1,070	0.21	−0.55 (−1.40, 0.30)	0.35	6	1.00	0
Sex	7	762/1,070	0.98	1.00 (0.82, 1.22)	3.44	6	0.75	0
BMI	6	532/840	0.09	0.45 (−0.07, 0.98)	5.78	5	0.33	13
Tumor size	6	741/1,047	0.43	−0.08 (−0.28, 0.12)	3.11	5	0.68	0

*RARN, robot-assisted radical nephrectomy; LRN, laparoscopic radical nephrectomy; BMI, body mass index; WMD, weighted*.

*mean difference; OR, odds ratio; CI, confidence interval*.

### Operating Time

**Figure 2:**
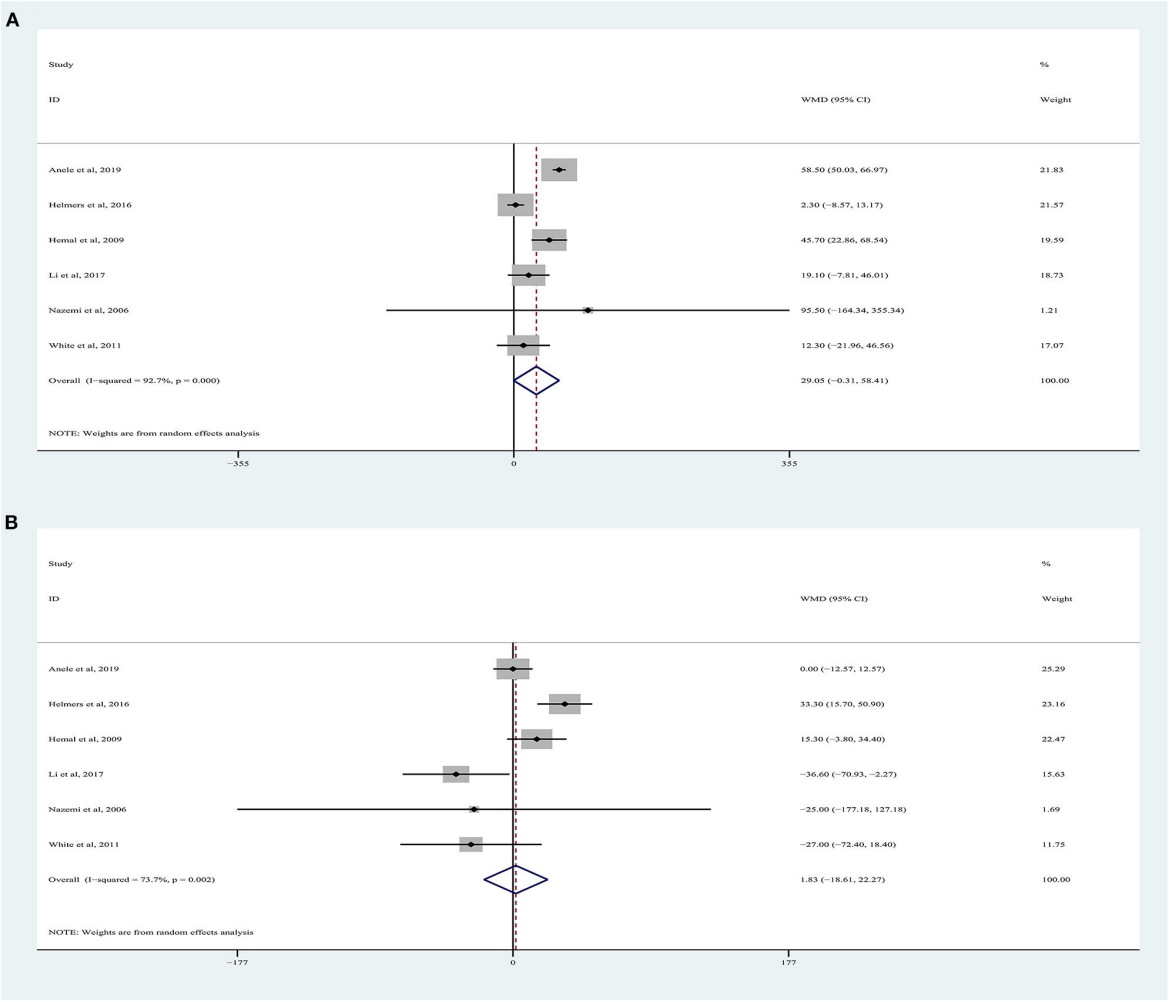
Forest plots of perioperative outcomes: **(A)** operating time, **(B)** estimated blood loss.

OT data were obtained from six studies (9, 18–22), totaling 1,372 patients (532 RARN vs. 840 LRN). The pooled analysis indicated no significant differences for OT between RARN and pure LRN (random-effects model: WMD, 29.05; 95% CI, −0.31, 58.41; *p* = 0.05; *I*^2^ = 93%), albeit at a greater heterogeneity (Figure 2A).

### Estimated Blood Loss

Six articles were included in the meta-analysis (9, 18–22), including 1,372 patients. Among them, 532 underwent RARN, and 840 underwent LRN (Figure 2B). Because higher heterogeneity was present, a random-effects model was used (*I*^2^ = 74%). The pooled outcome supported that EBL in the RARN group was similar to that in the standard LRN group (WMD, 1.83, 95% CI, −18.61, 22.27; *p* = 0.86).

### Length of Hospital Stay

**Figure 3:**
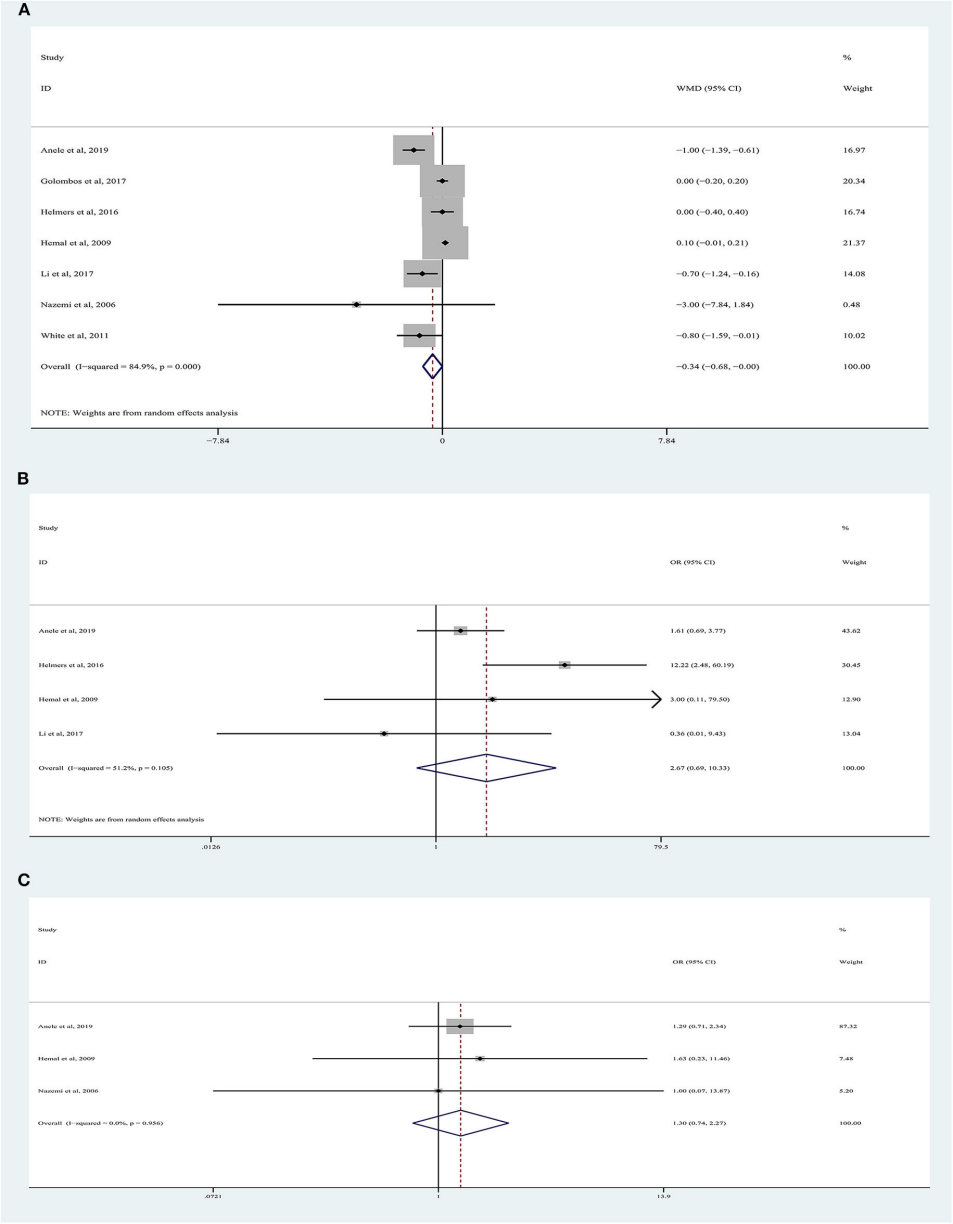
Forest plots of perioperative outcomes: **(A)** length of hospital stay, **(B)** transfusion rate, **(C)** conversion rate.

LOS data were reported in seven studies involving 1,832 patients (Figure 3A) (9, 10, 18–22), of which 762 underwent RARN and 1,070 underwent LRN. No significant difference was observed between the two groups regarding LOS (random-effects model: LOS, −0.34; 95% CI, −0.68, 0.00; *p* = 0.05; *I*^2^ = 85%), despite greater heterogeneity.

### Conversion Rate

There were 1,334 patients analyzed in four studies (19–22). The conversion rate was reported in 3.88% (20/516) of patients who underwent RARN and in 1.60% (13/813) of patients who underwent LRN (Figure 3B). Meta-analysis demonstrated that RARN offers a comparable conversion rate to LRN (random-effects model: WMD, 2.67; 95% CI, 0.69, 10.33; *p* = 0.16; *I*^2^ = 51%).

### Transfusion Rate

Three articles were analyzed (18, 19, 22). A total of 989 patients were included, of whom 425 underwent RARN and 564 underwent LRN (Figure 3C). The blood transfusion rate was similar between the two groups, and no heterogeneity was found (fixed-effects model: OR, 1.30; 95% CI, 0.74, 2.27; *p* = 0.36; *I*^2^ = 0%).

### Complications

**Figure 4:**
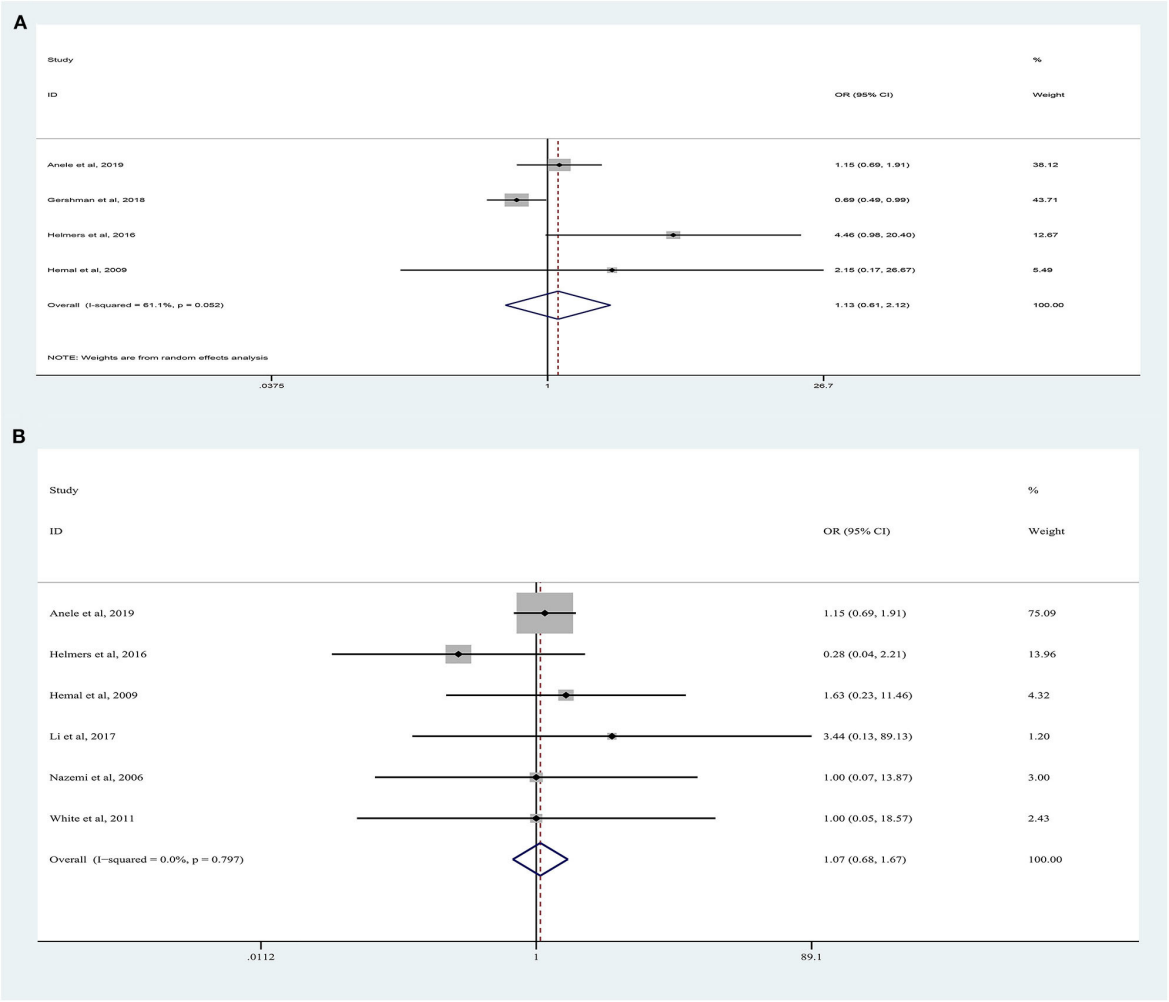
Forest plots of perioperative outcomes: **(A)** intraoperative complications, **(B)** postoperative complications.

Forest plots of perioperative complications are illustrated in Figure 4. There were no significant differences in intraoperative complications (random-effects model: OR, 1.13; 95% CI, 0.61, 2.12; *p* = 0.62; *I*^2^ = 61%) and postoperative complications (fixed-effects model: OR, 1.07; 95% CI, 0.68, 1.67; *p* = 0.62; *I*^2^ = 0%) between the two approaches.

### Sensitivity Analysis and Publication Bias

**Figure 5:**
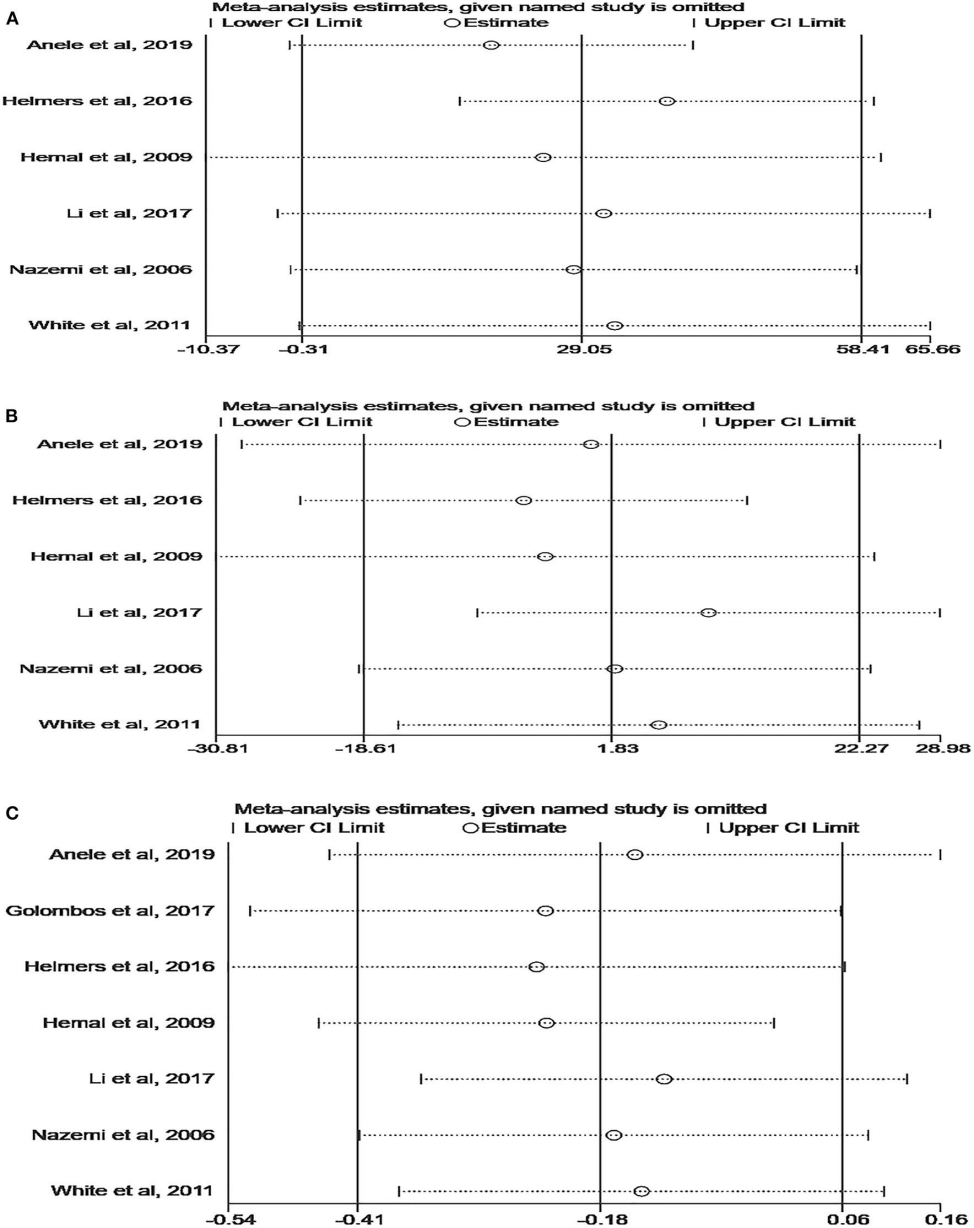
Sensitivity analysis of perioperative outcomes: **(A)** operating time, **(B)** estimated blood loss, **(C)** length of hospital stay.

Although the quality of the included studies was high (all scores were six or higher), some parameters were highly heterogeneous, such as OT, EBL, and LOS. A sensitivity analysis was performed on these parameters to improve the reliability of the analysis. Studies were removed one by one to recalculate the combined mean difference, and all the new pooled mean differences remained constant after deleting any study (Figure 5). The ROBINS-I tool was used to assess publication bias, and these results suggested that all comparative studies had a moderate risk of bias (9, 10, 18–22).

## Discussion

During the past decade, minimally invasive techniques have been used in the management of RCC (23). Both laparoscopic and robotic procedures are considered as alternatives to open surgery (7). In fact, the robotic procedure has higher definition displays and a shorter learning curve. Furthermore, RARN has a role in training for partial nephrectomy, in which robotic surgery offers some advantages compared to laparoscopy, such as finer manipulations and a greater range of motion, thus influencing the decision to continue a program of robotic surgery in which RN can be propaedeutic to partial nephrectomy.

Several studies have compared clinical parameters between RARN and standard LRN, but the advantage of these two technologies is still debatable (9, 12, 24). To the best of our knowledge, this is the first meta-analysis of reported comparative outcomes of RARN vs. LRN. The present study indicates that there were no significant differences between the two procedures in terms of OT, EBL, LOS, conversion rates, intraoperative complications, and postoperative complications. Therefore, RARN appears to be an effective and safe technique, although only the perioperative outcomes were compared.

In a retrospective cohort study conducted by Jeong et al. (11), the rate of prolonged OT (>4 h) in patients receiving RARN was higher than that for patients undergoing conventional LRN (11). Subsequently, using the multi-institutional renal masses database, Anele et al. (22) found that the duration of surgery for RARN was significantly longer than that for LRN, with a median OT increase of approximately 60 min (median = 185 and 126 min for RARN and LRN, respectively). Contrary to the results of both studies, our findings suggest that OT was comparable between RARN and LRN. Considering that greater heterogeneity was observed in the analysis (*I*^2^ = 93%), this result needs to be interpreted with caution.

In fact, there were different opinions on which method was superior in terms of OT. The duration of the operation may be related to the technical proficiency of the surgeon, and centers with less experience in robotic procedures may have longer OTs (25). Jaffe et al. (26) found that, after 180 cases, the OT of robotic surgery could be reduced from the initial 240 to 120 min. Similarly, Wolanski et al. (27) reported a steep improvement in operative duration as the robotic surgery experience increased. They concluded that the robot approach may have had significant advantages regarding console time compared to traditional laparoscopic surgery. As mentioned, differences in physician experience may lead to dramatic changes in surgical time, especially the time required for the suture part of the procedure. Moreover, different definitions of surgical time in the included studies may contribute to variations in results.

Our meta-analysis suggested that the EBL was similar between RARN and LRN (*p* = 0.86), which is consistent with prior studies (9, 19, 22). Helmers et al. (20) performed a retrospective study with 319 cases (243 RARN and 76 LRN). They described that the RARN group had a greater EBL than the LRN group (median = 100 vs. 50 mL, *p* < 0.05). However, Li et al. (21) found that the mean EBL was significantly lower for RARN (53.8 mL) than for standard LRN (90.4 mL). They explained that this difference is due to the operator's ability to accurately separate renal arteries and veins in the high-definition field of view provided by the robot, which can better protect blood vessels and reduce blood loss during surgery.

Of note, all included comparative studies reported LOS, and the pooled results demonstrated that no significant difference was found in LOS between the two technologies. One prospective study, instead, reported the LOS in RARN was significantly shorter than that in LRN (4.4 vs. 5.1 days, *p* < 0.05) (21). Anele et al. (22) found RARN had a lower median LOS (3 days in the RARN group vs. 5 days in the LRN group, *p* < 0.001). In addition, the flexible operation of the robotic method may increase the surgeon's confidence in the quality of the anastomosis. This may shorten the retention time of the drainage tube, thereby reducing the LOS. Heterogeneity was also found in the analysis for this outcome (*I*^2^ = 85%); however, no change in heterogeneity was observed after deleting any study, and the merger results were still not significant, which undoubtedly increased the credibility of our study.

Another interesting finding was the similar conversion rate for the RARN and LRN groups, according to the present study. This is different from the data reported Helmers et al. (20). Their results indicated that RARN was associated with a higher conversion rate than traditional LRN (10.3 vs. 1.0%, *p* < 0.01). A higher conversion rate in RARN was caused by bleeding and dense adhesions, but the median tumor size of the converted RRN cases was 9 cm. A larger tumor makes the dissection of kidney blood vessels more difficult, increasing the risk of causing serious vascular damage (19). Furthermore, confounding factors that are difficult to analyze may also be important, such as kidney anatomical variation and surgeon technical proficiency. In terms of blood transfusion rate, there was no steep difference between RARN and LRN, which is highly compatible with the results of previous comparative studies (18, 19, 22). However, prospective randomized controlled trials are needed to confirm our findings.

Complications are an important parameter for estimating the safety of surgical techniques. Our study suggested that RARN had similar intraoperative and postoperative complications as those of LRN. However, using the Nationwide Inpatient Sample data from 2010 to 2013, Gershman et al. (12) found that RARN was associated with lower perioperative morbidity (20.4 vs. 27.2%, *p* < 0.001), and surgeons with extensive RARN experience can diminish or avoid collateral injuries during surgery. Given the potential confounding factors, including patient characteristics, tumor complexity, and hospital location, this issue requires further investigation.

Since the introduction of RARN, one of the major concerns has been the high cost, which was supposed to be the chief obstruction of the broad implementation of this technology. In a cost comparison analysis, Jeong et al. (11) reported that the average 90-day direct hospital costs for RARN were significantly higher than those for LRN (US $19,530 vs. $16,851, *p* = 0.004). Similarly, Gershman et al. (12) also found that RARN had higher total hospital charges (US $16,207 vs. $15,037, *p* < 0.001). Interestingly, in the single-institution study conducted by Helmers et al. (20), there was no significant difference in total inpatient costs between the procedures (median = US $14,913 vs. $16,265, *p* = 0.171). As discussed by Kates et al. (28), the shorter LOS of the RARN procedure may save hospital charges; however, additional prospective studies are needed to explain this issue. In addition, the cost variations may depend on the size, location, and profit status of the medical center (29).

Limited studies have reported direct comparisons between RARN and standard LRN regarding oncologic outcomes. Golombos et al. (10) reported no significant differences in overall and cancer-specific survival between RARN and LRN at midterm follow-up. According to a comparative study, the median follow-up for RARN and LRN was 15 and 20 months, respectively, and no difference was noted in overall survival between the two groups (22). However, RARN was associated with a higher positive margin rate and greater risk of recurrence or metastases than LRN (all *p* < 0.01). As described, the higher staging and grading of malignancies in the RARN group may contribute to this result. Chopra et al. (30) reported results of RARN in patients with RCC with inferior vena cava tumor thrombus. The authors reported that no patient died during a median follow-up of 16 months (range = 12–39 months). Thus, RARN appears to have acceptable short-term oncologic outcomes, including locally advanced/progressed RCC, and in the case of renal cancer with inferior vena cava tumor thrombosis (7, 30, 31).

Our study is of clinical value as the first meta-analysis to directly compare the perioperative outcomes of RARN and LRN in patients with RCC. However, there are some limitations to the present analysis. First, most of the included studies had a retrospective design, and the sample size of some studies was small. Therefore, the level of evidence in this study was low. Second, some outcomes were heterogeneous, including OT, EBL, and LOS. Sensitivity analysis explained partial heterogeneity, but subgroup analysis was not available to further analyze other confounding factors with limited data. Considering these, our findings should be interpreted with caution. Third, many studies reported a shorter follow-up period, and only two studies had a median follow-up of more than 1 year. As a result, the oncologic outcomes of the two technologies cannot be assessed. Finally, a cost analysis was not evaluated because of lack of data; indeed, cost is the main factor restricting the implementation of robotics.

In summary, the study demonstrated that RARN was not superior to LRN in patients with RCC, in terms of perioperative outcomes. Before establishing final clinical recommendations, high-quality prospective large-scale randomized controlled trials with long-term follow-up are needed.

## Data Availability Statement

All datasets generated for this study are included in the article/ supplementary material.

## Author Contributions

YL and QW: conception and design. JL, LP, DC, and BC: acquisition of data and critical revision of the manuscript for important intellectual content. JL, LP, and HG: analysis and interpretation of data. JL and LP: drafting of the manuscript. YL: supervision. All authors: read and agree to the published version of the manuscript.

## Conflict of Interest

The authors declare that the research was conducted in the absence of any commercial or financial relationships that could be construed as a potential conflict of interest.
